# Comparison of behavioral activation/inhibition systems, emotional regulation difficulties, and selective attention in adolescents with and without parents

**DOI:** 10.3389/fpsyg.2023.1212187

**Published:** 2023-09-01

**Authors:** Zohreh Sadeghzadeh, Fariborz Bagheri

**Affiliations:** Department of Psychology, Islamic Azad University, Science and Research Branch, Tehran, Iran

**Keywords:** behavioral activation system, behavioral inhibition system, emotion dysregulation, selective attention, parents, adolescence

## Abstract

Existing literature has established a relationship between adverse childhood experiences and negative outcomes in cognitive and affective functioning. However, further research is needed to thoroughly understand and validate these findings. In this regard, the current study aimed to compare behavioral activation/inhibition systems, emotional regulation difficulties, and selective attention in adolescents with and without parents. A sample of 70 adolescents (M age = 16.36, *SD* = 1.09, 48.57% female) with caretakers from schools and 55 parentless adolescents (M age = 16.58, *SD* = 1.28, 52.00% female) from orphanage centers in Tehran were recruited and completed the measures tapping behavioral activation/inhibition systems, emotion dysregulation difficulties, and selective attention. The results revealed that parentless adolescents exhibited significantly higher levels of behavioral activation/inhibition scores, emotion regulation difficulties, and impaired selective attention. These findings suggest that the absence of parents during the early years of life may have detrimental effects on behavioral inhibition and activation systems, emotional regulation abilities, and selective attention capabilities. The implications of these findings are further discussed.

## Introduction

Early childhood development is known to be heavily influenced by environmental factors, such as family, caregivers, environment, and culture. The family environment, in particular, is considered the most natural and appropriate place for forming and stabilizing individuals’ cognitive and emotional skills (e.g., Bellis et al., 2018). Optimal growth and development in children are contingent on the provision of supportive environmental stimuli and a strong relationship with primary caregivers (Schore, 2001). Unfortunately, in contemporary society, a significant number of children and adolescents are deprived of parental care and effective parent–child relationship, which can include a wide range of children, such as those in boarding schools, homeless children, children of single parents, and children from displaced and homeless families who lack effective guardianship (e.g., Edwards, 2020). The support of parents and caregivers is crucial in promoting the health and well-being of children. Lack of parental love and attention, and failure to meet developmental needs such as belonging, admiration, and affection, can lead to a higher prevalence of psychological problems, behavioral disorders, aggression, anxiety, depression, and a tendency to use drugs (Eslami et al., 2013). Studies have shown that parentless children perform significantly lower in developmental fields, particularly in behavioral tests, intelligence, and speech abilities, compared to children who received proper parental care. Delays in cognitive and social development can result in academic and learning problems in these teenagers. Deprivation and early exposure to care centers or orphanages not only affect children’s social and emotional abilities but also have negative consequences in adolescence and adulthood, such as cognitive defects, high sensitivity to stress, and vulnerability to diseases (e.g., Gunnar and Fisher, 2006; McLaughlin et al., 2011, 2014). Given the wide range of behavioral, emotional, and cognitive problems among children and teenagers living in welfare organizations and care centers (e.g., orphanages), it is crucial to investigate the different dimensions of impairments in these areas in this group.

The development of approach and avoidance traits in childhood is crucial to the manifestation of individual differences in motivated behaviors and emotional experiences throughout life. Theories of personality and temperament have converged on the significance of the Behavioral Approach System (BAS) and the Behavioral Inhibition System (BIS; Fowles, 1980; Gray, 1990; Elliot and Thrash, 2002). The BAS mediates responses to positive reinforcement to promote reward-seeking behaviors, whereas the BIS responds to potentially aversive stimuli to inhibit behaviors that could lead to harmful outcomes. Studies have shown that an overactive BAS is associated with an increased risk for impulse control, substance use, and attention-deficit/hyperactivity disorders. On the other hand, extreme BIS sensitivity is linked to the development of anxiety disorders, depression, and psychosomatic illnesses (van den Berg et al., 2011; Becker et al., 2013; Park et al., 2013). As the field of temperament research has advanced, newer theories have emerged that complement and expand upon the BAS and BIS constructs. One such noteworthy framework is proposed by Rothbart and Hwang (2005) and Rothbart (2007), introducing several crucial concepts related to individual differences in temperament. Rothbart’s temperament theory emphasizes the multidimensional nature of temperament, considering various components that influence an individual’s responses to the environment. One of these components is “effortful control,” which pertains to an individual’s ability to regulate their responses, including attentional control, inhibitory control, and perceptual sensitivity. This aspect of temperament has significant implications for an individual’s approach or avoidance tendencies in various situations. Furthermore, Rothbart’s theory incorporates the concept of “negative affectivity,” akin to the notion of behavioral inhibition. Negative affectivity refers to individual differences in the tendency to experience negative emotions and the intensity of these emotional responses, impacting an individual’s avoidance behaviors (Rothbart and Hwang, 2005; Rothbart, 2007).

While BIS and BAS systems have a strong genetic basis, studies suggest that both systems are influenced by environmental factors(Ide et al., 2020). Hence, it is reasonable to expect that adverse childhood experiences, which are commonly encountered by parentless children, may impact the functioning of BIS and BAS systems.

In addition, the family context and parents play a crucial role in the development of children and adolescents’ emotion regulation (ER) abilities. ER refers to the ability to adjust emotional arousal and accomplish goal-directed behaviors regardless of emotional state; deficits in ER or emotion dysregulation (ED) lead to difficulties in monitoring, evaluating, or adjusting emotional reactions (Gratz and Roemer, 2004; Gross, 2013). Parenting practices related to emotion management and the emotional climate of the family affect ER. Children learn ER skills through observation, modeling, and social referencing. Factors like attachment relationships, family expressiveness, and parenting style impact the development of ER skills in children (For a review, see Morris et al., 2007). More specifically, children in orphanages suffer from ER problems which could be related to the lack of consistent and nurturing caregiving. Children who have experienced early life adversity, such as separation from their parents, are more likely to develop ER difficulties. In orphanages, children may not receive the individualized attention and emotional support needed to develop healthy coping skills. Additionally, the stressful and unpredictable environment of an orphanage can exacerbate ED, which can lead to a range of negative outcomes such as behavioral problems, academic difficulties, and mental health issues (Bos et al., 2009; McLaughlin et al., 2011; Cullen et al., 2014). Furthermore, research suggests that early life adversity can lead to alterations in neural pathways and structures involved in attention and cognitive control, which can result in difficulties in selective attention among individuals who have experienced childhood adversity (McLaughlin et al., 2014). These alterations in brain structure and function can result in attentional biases toward threat-related stimuli, as well as difficulties in disengaging attention from negative stimuli (Pollak et al., 2010). Also, given the higher levels of negative emotions that unsupervised youngsters experience, these individuals selectively allocate visual attention toward the locus of threatening information. This can result in a build-up of attentional resources towards threatening stimuli, leaving little capacity for the individual to attend to neutral or ongoing tasks, ultimately resulting in a reduction of the individual’s attentional resources for the performance of such tasks (e.g., Rudaizky et al., 2021).

The above literature review suggests that inadequate parental care can have negative effects on individuals’ brain activation/inhibition systems, emotional regulation abilities, and selective attention. However, empirical studies are needed to further explore this topic. Children and teenagers in welfare organizations or orphanages often lack consistent and nurturing caregivers, and comparing their emotional regulation abilities and cognitive development to those with parents could help expand the existing literature. To fill this gap, this study aims to compare behavioral activation/inhibition systems, emotional regulation difficulties, and selective attention in adolescents with and without parents.

## Method

### Participants and procedure

The sample consisted of 70 adolescents (aged 15–18, M age = 16.36, *SD* = 1.09, 48.57% female) with parents recruited through convenience sampling from schools in Tehran, and 55 parentless adolescents (aged 15–18, M age = 16.58, *SD* = 1.28, 52.00% female) from orphanage centers in Tehran recruited through purposeful sampling. The inclusion criteria for all participants consisted of interest and willingness to participate in the study. For the orphanage sample, inclusion criteria comprised a residency in orphanage centers of Tehran province for more than 1 year; exclusion criteria were a history of substance abuse and other serious mental disorders assessed through file review and clinical interview. The ethics committee of *Islamic Azad University, Science and Research Branch* approved the study, and participants and their guardians were informed about the study’s goals and voluntary nature before providing signed informed consent. The same procedure was followed for the orphanage sample, with their supervisors in the centers also informed about the study’s aims. All participants were surveyed, except for those who declined. Participants were informed again about the confidentiality of their information and were asked to complete the questionnaires under the supervision of a specially trained research assistant (a master-level student).

### Measures

#### The behavioral inhibition/behavioral activation system scales (BIS/BAS scales)

The BIS/BAS scales (Carver and White, 1994) is a 20-item self-report questionnaire designed to assess the extent to which an individual is sensitive to rewards and punishments. The BIS/BAS Scale measure both the Behavioral Inhibition System (BIS) and three types of Behavioral Activation System (BAS) reactivity, which include Drive, Reward Responsiveness, and Fun Seeking. Items are rated on a Likert-type scale ranging from 1 (*“I strongly agree”*) to 4 (*“I strongly disagree”*), and respondents rate the extent to which they agree or disagree with each statement. The Persian version of the BIS/BAS Scale yielded sound psychometric properties (Amiri and Hassani, 2016).

### Difficulties in emotion regulation scale

The DERS (Gratz and Roemer, 2004) is a 36-item self-report questionnaire that assesses emotion dysregulation. The DERS items load on six subscales, including Lack of Emotional Awareness (6 items), Lack of Emotional Clarity (5 items), Difficulties Controlling Impulsive Behaviors When Distressed (6 items), Difficulties Engaging in Goal-Directed Behavior When Distressed (5 items), Nonacceptance of Negative Emotional Responses (6 items), and Limited Access to Effective ER Strategies (8 items). Participants rate items on a 5-point scale ranging from 1 (*“almost never”*) to 5 (*“almost always”*). A total score is obtained by summing all items. The internal consistency and validity of the Persian version of DERS were supported with the Iranian sample in previous studies (Besharat and Bazzazian, 2013; Vafa et al., 2021).

### D2 attention test

The D2 was developed by Brickenkamp and Zillmer (1998) and is a widely used one-page paper-and-pencil test that measures selective attention and cognitive performance. It consists of 14 rows (trials) with 47 randomly mixed “p” and “d” letters per row, for a total of 658 letters, with the target symbol being a “d” with two dashes. Participants are instructed to cancel out as many target symbols as possible within a 20-s time limit, moving from left to right. In the current study, the following D2 subscores were used: total number (TN), omissions (E1), commissions (E2), and concentration performance (CP). TN is a quantitative measure of performance, while E1 and E2 reflect different types of errors. CP is derived from the number of correctly crossed-out relevant items minus the number of E2 errors.

### Data analyses

In the current study, data entry and statistical analyses were performed using SPSS 20 software. The normality of the distribution for study variables was tested using the Kolmogorov–Smirnov test, and the results supported the normality of the data (*p* > 0.05). Next, independent *t*-tests were then used to compare the two groups on the study variables, along with examining Cohen’s *d* as a measure of effect size, with a value of 0.20 denoting a small, 0.50 a moderate, and 0.80 a large effect with effect sizes to estimate the magnitude of the differences (Cohen, 2013). A significance level (*p*-value) of less than 0.05 was predetermined to indicate statistically significant results.

## Results

As shown in Table 1, independent *t*-tests results showed that the parentless adolescents group scored significantly higher in BIS and BAS and its subscales of drive, reward responsiveness, and fun seeking (*p* < 0.001 to 0.013; *d* = 0.33 to 1.07). Similarly, results were indicative of significantly higher DERS and its all but non-acceptance of negative emotional responses subscales for the parentless adolescents group (*p* < 0.001 to 0.03; *d* = 0.45 to 0.85). Table 1 also shows that parentless adolescents performed significantly poor in D2 attention test scores of the total number (TN), omissions (E1), commissions (E2), and concentration performance (CP; *p* < 0.001 to 0.02; *d* = 0.46 to 0.88).

**Table 1:** Comparisons of study variables across the two groups.

Supervised (*n* = 70)		Unsupervised (*n* = 55)	Group comparison		
	M (*SD*)	M (*SD*)	*t*	*p*	*d*
Behavioral inhibition system	19.58 (2.51)	21.99 (3.48)	4.18	0.001	0.79
Behavioral activation system	32.38 (6.17)	38.03 (7.92)	5.62	0.001	1.07
Drive	11.40 (1.89)	13.52 (2.53)	5.01	0.001	0.94
Reward responsiveness	9.68 (2.08)	12.05 (2.99)	4.83	0.001	0.92
Fun seeking	11.30 (2.20)	12.46 (2.40)	2.70	0.013	0.33
DERS total	77.32 (17.48)	93.60 (20.48)	4.55	0.001	0.85
Goal	12.90 (4.94)	15.01 (4.40)	2.45	0.03	0.45
Impulse	10.24 (4.90)	13.56 (5.64)	3.35	0.001	0.62
Non-acceptance	13.44 (5.37)	14.21 (5.88)	0.73	0.93	0.14
Lack awareness	14.67 (4.62)	18.23 (5.45)	3.75	0.001	0.70
Strategies	15.47 (5.58)	19.02 (6.38)	3.16	0.001	0.59
Clarity	10.60 (3.78)	13.57 (4.82)	3.63	0.001	0.68
Total number (TN)	449.31 (95.03)	403.09 (102.33)	2.51	0.02	0.46
Omissions (E1)	37.79 (26.21)	59.11 (50.56)	2.73	0.01	0.52
Commissions (E2)	4.29 (12.43)	12.91 (19.27)	2.77	0.01	0.53
Concentration performance (CP)	156.54 (33.42)	123.85 (40.41)	4.68	0.001	0.88

DERS = Difficulties in Emotion Regulation Scale; Goal = Difficulties Engaging in Goal-Directed Behavior; Impulse = Difficulties Controlling Impulsive Behaviors; Accept = Non-acceptance of Negative Emotional Responses; Strategy = Limited Access to Emotion Regulation strategies; Clarity = lack of emotional clarity; M = Mean; *SD* = Standard deviation; *t* = Independent Student’s *t*-test; *d* = Cohen’s *d*.

## Discussion

This study aims to compare behavioral activation/inhibition systems, emotional regulation difficulties, and selective attention in adolescents with and without parents. The results showed that parentless adolescents had significantly higher levels of BIS/BAS scores, emotion regulation difficulties, and impaired selective attention. Overall, in line with the literature, our findings suggest that the absence of parents during the early years of life may have detrimental effects on levels of behavioral inhibition and activation system, development of emotional regulation abilities, and selective attention capability. Environmental factors, such as family dynamics, caregiving practices, surroundings, and cultural influences, play a crucial role in shaping early childhood development. Notably, the family environment is widely recognized as the most natural and vital context for fostering cognitive and emotional skills in young individuals (Bellis et al., 2018). Optimal growth and development in children hinge upon the provision of supportive environmental stimuli and the establishment of a secure relationship with primary caregivers (e.g., Schore, 2001; Bellis et al., 2018).

With respect to our findings that show increased levels of BIS and BAS among parentless adolescents, it is important to highlight that although there is solid evidence supporting the genetic basis of these systems, research suggests that environmental factors also play a significant role in shaping BIS and BAS responses (e.g., Ide et al., 2020). For instance, as a common phenomenon among parentless children, adverse childhood experiences could potentially affect the development of both systems. Adverse childhood experiences may result in diminished BIS activation due to chronic stress or trauma during childhood, which can desensitize the BIS and reduce responsiveness to threat cues, leading to decreased cautiousness. Likewise, adverse childhood experiences may also lead to heightened BAS activation in adolescence. Adversities such as neglect, abuse, or exposure to violence can disrupt the development of self-regulation and emotion-regulation skills, resulting in increased reward sensitivity, and sensation-seeking behaviors, which are associated with higher BAS activation (e.g., Nelson, 2000; Pechtel and Pizzagalli, 2011; Tottenham, 2014).

Our findings also revealed that parentless adolescents had higher levels of emotion dysregulation (ED). The way parents manage emotions and create an emotional climate within families has a significant impact on the development of emotion regulation (ER) skills in children. Children learn ER skills through observation, modeling, and social referencing, and factors such as attachment relationships, family expressiveness, and parenting style can shape the development of these skills in children (Morris et al., 2007). Specifically, children who have experienced early life adversity, such as separation from their parents as observed in orphanages, are more likely to face difficulties with ER. The lack of consistent and nurturing caregiving in orphanages can impede the development of healthy coping skills, as children may not receive the individualized attention and emotional support that is necessary. Furthermore, the stressful and unpredictable environment of an orphanage can worsen emotion dysregulation, exacerbating the challenges faced by these children (Bos et al., 2009; McLaughlin et al., 2011; Cullen et al., 2014).

Finally, our findings revealed that parentless adolescents had significantly higher levels of selective attention problems. This is supported by existing literature indicating that adverse childhood experiences are associated with deficits in various cognitive functions, including cognitive performance, memory, and executive functioning (for a review, see Pechtel and Pizzagalli, 2011). Additionally, unsupervised youngsters who experience higher levels of negative emotions may selectively allocate visual attention toward threatening information. This can lead to an accumulation of attentional resources towards threatening stimuli, leaving limited capacity for attending to neutral or ongoing tasks, ultimately resulting in reduced attentional resources for task performance (Rudaizky et al., 2021).

Our findings should be interpreted in the context of some limitations. First, the cross-sectional design of this study precludes establishing a cause-and-effect relationship between the variables. Longitudinal studies that track individuals over time would be needed to establish causality and gain a deeper understanding of the developmental trajectory of parentless adolescents. Second, the relatively small sample size of this study and the inability to conduct separate analyses across gender groups may limit the generalizability of the findings. Replicating these findings in larger samples that include diverse populations would enhance the external validity of the results and increase confidence in their generalizability. Third, the use of self-report measures to assess the variables of interest may introduce biases and may not fully capture the complexities of the constructs being measured. Incorporating multiple assessment methods, such as behavioral observations or physiological measures, in future studies would provide a more comprehensive understanding of the phenomena under investigation and strengthen the validity of the findings. Finally, we did not incorporate measurements related to parenthood, time spent away from parents, and time spent in residence during the early years of life in our study. We recommend that future research thoroughly investigate the influence of these critical factors to further examine the robustness of our findings.

Overall, our results emphasize the significance of parenthood during the early years of life and suggest the potential need for targeted interventions to address behavioral, emotional, and cognitive difficulties in parentless adolescents. Further research in this area is warranted to better understand the underlying mechanisms and develop effective interventions for this vulnerable population. These findings highlight the importance of addressing the unique needs of parentless adolescents and ensuring that they receive appropriate support during their early years of life.

## Data availability statement

The raw data supporting the conclusions of this article will be made available by the authors, without undue reservation.

## Ethics statement

The studies involving humans were approved by The ethics committee of Islamic Azad University, Science and Research Branch. The studies were conducted in accordance with the local legislation and institutional requirements. Written informed consent for participation in this study was provided by the participants’ legal guardians/next of kin.

## Author contributions

ZS: gathered the data, performed data analyses, and prepared the manuscript. FB: supervised the study and reviewed and revised the manuscript. All authors contributed to the article and approved the submitted version.

## Conflict of interest

The authors declare that the research was conducted in the absence of any commercial or financial relationships that could be construed as a potential conflict of interest.

## Publisher’s note

All claims expressed in this article are solely those of the authors and do not necessarily represent those of their affiliated organizations, or those of the publisher, the editors and the reviewers. Any product that may be evaluated in this article, or claim that may be made by its manufacturer, is not guaranteed or endorsed by the publisher.

## References

[ref1] Amiri S. Hassani J. (2016). Assessment of psychometric properties of behavioral activation and behavioral inhibition systems scale associated with impulsivity and anxiety [research]. Razi J. Med. Sci. 23, 68–80.

[ref2] Becker S. P. Fite P. J. Garner A. A. Greening L. Stoppelbein L. Luebbe A. M. (2013). Reward and punishment sensitivity are differentially associated with ADHD and sluggish cognitive tempo symptoms in children. J. Res. Pers. 47, 719–727. doi: 10.1016/j.jrp.2013.07.001

[ref3] Bellis M. A. Hughes K. Ford K. Hardcastle K. A. Sharp C. A. Wood S.. (2018). Adverse childhood experiences and sources of childhood resilience: a retrospective study of their combined relationships with child health and educational attendance. BMC Public Health 18:792. doi: 10.1186/s12889-018-5699-8, PMID: 29940920 PMC6020215

[ref4] Besharat M. A. Bazzazian S. (2013). Psychometric properties of the cognitive emotion regulation questionnaire in a sample of Iranian population. Nurs. Midwifery J. 24, 61–70.

[ref5] Bos K. J. Fox N. Zeanah C. H. Nelson Iii C. A. (2009). Effects of early psychosocial deprivation on the development of memory and executive function. Front. Behav. Neurosci. 3:16. doi: 10.3389/neuro.08.016.2009, PMID: 19750200 PMC2741295

[ref6] Brickenkamp R. Zillmer E. (1998). Test d2: concentration-endurance test. Gottingen Ger. CJ Hogrefe.

[ref7] Carver C. S. White T. L. (1994). Behavioral inhibition, behavioral activation, and affective responses to impending reward and punishment: the BIS/BAS scales. J. Pers. Soc. Psychol. 67, 319–333. doi: 10.1037/0022-3514.67.2.319

[ref8] Cohen J. (2013). Statistical power analysis for the behavioral sciences. New York: Routledge.

[ref9] Cullen K. R. Westlund M. K. Klimes-Dougan B. Mueller B. A. Houri A. Eberly L. E.. (2014). Abnormal amygdala resting-state functional connectivity in adolescent depression. JAMA Psychiatry 71, 1138–1147. doi: 10.1001/jamapsychiatry.2014.1087, PMID: 25133665 PMC4378862

[ref10] Edwards E. J. (2020). Young, black, successful, and homeless: examining the unique academic challenges of black students who experienced homelessness. J. Child. Poverty 26, 125–149. doi: 10.1080/10796126.2020.1776688

[ref11] Elliot A. J. Thrash T. M. (2002). Approach-avoidance motivation in personality: approach and avoidance temperaments and goals. J. Pers. Soc. Psychol. 82, 804–818. doi: 10.1037/0022-3514.82.5.804, PMID: 12003479

[ref12] Eslami R. Hashemian P. Jarahi L. Modarres Gharavi M. (2013). Effectiveness of group reality therapy on happiness and quality of life in unsupervised adolescents in Mashhad. Med. J. Mashhad Univ. Med. Sci. 56, 300–306. doi: 10.22038/mjms.2013.1958

[ref13] Fowles D. C. (1980). The three arousal model: implications of Gray's two-factor learning theory for heart rate, Electrodermal activity, and psychopathy. Psychophysiology 17, 87–104. doi: 10.1111/j.1469-8986.1980.tb00117.x, PMID: 6103567

[ref14] Gratz K. L. Roemer L. (2004). Multidimensional assessment of emotion regulation and dysregulation: development, factor structure, and initial validation of the difficulties in emotion regulation scale. J. Psychopathol. Behav. Assess. 26, 41–54. doi: 10.1023/B:JOBA.0000007455.08539.94

[ref15] Gray J. A. (1990). Brain systems that mediate both emotion and cognition. Cognit. Emot. 4, 269–288. doi: 10.1080/02699939008410799

[ref16] Gross J. J. (2013). Handbook of emotion regulation. New York: Guilford publications.

[ref17] Gunnar M. R. Fisher P. A. (2006). Bringing basic research on early experience and stress neurobiology to bear on preventive interventions for neglected and maltreated children. Dev. Psychopathol. 18, 651–677. doi: 10.1017/S0954579406060330 17152395

[ref18] Ide J. S. Li H.-T. Chen Y. Le T. M. Li C. S. P. Zhornitsky S.. (2020). Gray matter volumetric correlates of behavioral activation and inhibition system traits in children: an exploratory voxel-based morphometry study of the ABCD project data. NeuroImage 220:117085. doi: 10.1016/j.neuroimage.2020.117085, PMID: 32592852 PMC7572877

[ref19] McLaughlin K. A. Fox N. A. Zeanah C. H. Nelson C. A. (2011). Adverse rearing environments and neural development in children: the development of frontal electroencephalogram asymmetry. Biol. Psychiatry 70, 1008–1015. doi: 10.1016/j.biopsych.2011.08.006, PMID: 21962332 PMC3207006

[ref20] McLaughlin K. A. Sheridan M. A. Lambert H. K. (2014). Childhood adversity and neural development: deprivation and threat as distinct dimensions of early experience. Neurosci. Biobehav. Rev. 47, 578–591. doi: 10.1016/j.neubiorev.2014.10.012, PMID: 25454359 PMC4308474

[ref21] Morris A. S. Silk J. S. Steinberg L. Myers S. S. Robinson L. R. (2007). The role of the family context in the development of emotion regulation. Soc. Dev. 16, 361–388. doi: 10.1111/j.1467-9507.2007.00389.x, PMID: 19756175 PMC2743505

[ref22] Nelson C. A. (2000). The effects of early adversity on neurobehavioral development. Hove, East Sussex, UK: Psychology Press.

[ref23] Park S. M. Park Y. A. Lee H. W. Jung H. Y. Lee J.-Y. Choi J.-S. (2013). The effects of behavioral inhibition/approach system as predictors of internet addiction in adolescents. Personal. Individ. Differ. 54, 7–11. doi: 10.1016/j.paid.2012.07.033

[ref24] Pechtel P. Pizzagalli D. A. (2011). Effects of early life stress on cognitive and affective function: an integrated review of human literature. Psychopharmacology 214, 55–70. doi: 10.1007/s00213-010-2009-2, PMID: 20865251 PMC3050094

[ref25] Pollak S. D. Nelson C. A. Schlaak M. F. Roeber B. J. Wewerka S. S. Wiik K. L.. (2010). Neurodevelopmental effects of early deprivation in postinstitutionalized children. Child Dev. 81, 224–236. doi: 10.1111/j.1467-8624.2009.01391.x, PMID: 20331664 PMC2846096

[ref26] Rothbart M. K. (2007). Temperament, development, and personality. Curr. Dir. Psychol. Sci. 16, 207–212. doi: 10.1111/j.1467-8721.2007.00505.x

[ref27] Rothbart M. K. Hwang J. (2005). “Temperament and the Development of Competence and Motivation,” in Handbook of competence and motivation. eds. A. J. Elliot and C. S. Dweck (New York: Guilford Publications), 167–184.

[ref28] Rudaizky D. Bebbington K. Davis E. A. Radcliffe W. MacLeod C. Hunt A.. (2021). Selective attention to threat, anxiety and glycaemic management in adolescents with type 1 diabetes. Comprehen. Psychoneuro. 7:100065. doi: 10.1016/j.cpnec.2021.100065, PMID: 35757060 PMC9216651

[ref29] Schore A. N. (2001). Effects of a secure attachment relationship on right brain development, affect regulation, and infant mental health. Infant Ment. Health J. 22, 7–66. doi: 10.1002/1097-0355(200101/04)22:1<7::AID-IMHJ2>3.0.CO;2-N

[ref30] Tottenham N. (2014). “The importance of early experiences for neuro-affective development” in The neurobiology of childhood. eds. Andersen S. L. Pine D. S. (Berlin Heidelberg: Springer), 109–129. 10.1007/7854_2013_254 PMC4021037 24264369

[ref31] Vafa Z. Azizi M. Elhami Athar M. (2021). Predicting academic alienation from emotion dysregulation, social competence, and peer relationships in school-attending girls: a multiple-regression approach. Front. Psychol. 12:755952. doi: 10.3389/fpsyg.2021.755952, PMID: 35035367 PMC8759297

[ref32] van den Berg L. Pieterse K. Malik J. A. Luman M. Willems van Dijk K. Oosterlaan J.. (2011). Association between impulsivity, reward responsiveness and body mass index in children. Int. J. Obes. 35, 1301–1307. doi: 10.1038/ijo.2011.116 21694699

